# The 1-minute sit-to-stand test to detect desaturation during 6-minute walk test in interstitial lung disease

**DOI:** 10.1038/s41533-022-00268-w

**Published:** 2022-01-27

**Authors:** Keiji Oishi, Kazuto Matsunaga, Maki Asami-Noyama, Tasuku Yamamoto, Yukari Hisamoto, Tetsuya Fujii, Misa Harada, Junki Suizu, Keita Murakawa, Ayumi Chikumoto, Kazuki Matsuda, Haruka Kanesada, Yujiro Kikuchi, Kazuki Hamada, Sho Uehara, Ryo Suetake, Syuichiro Ohata, Yoriyuki Murata, Yoshikazu Yamaji, Kenji Sakamoto, Kosuke Ito, Hisayuki Osoreda, Nobutaka Edakuni, Tomoyuki Kakugawa, Tsunahiko Hirano, Masafumi Yano

**Affiliations:** 1 Department of Medicine and Clinical Science, Graduate School of Medicine, Yamaguchi University, Ube, Japan; 2 Department of Respiratory Medicine and Infectious Disease, Graduate School of Medicine, Yamaguchi University, Ube, Japan; 3 Department of Respiratory Medicine, National Hospital Organization Yamaguchi-Ube Medical Center, Ube, Japan; 4 Department of Pulmonology and Gerontology, Graduate School of Medicine, Yamaguchi University, Ube, Japan

**Keywords:** Respiratory signs and symptoms, Physical examination

## Abstract

Although the 6 min walk test (6MWT) is well-established for assessing desaturation in patients with interstitial lung disease (ILD), it cannot be easily performed in primary healthcare settings. This retrospective observational study aimed to evaluate the usefulness of the 1 min sit-to-stand test (1STST) for assessing desaturation during 6MWT in ILD patients with normal resting blood oxygen levels. We included 116 patients, and the pulse oxygen saturation (SpO_2_) for both methods was analyzed. The SpO_2_ nadir during the 1STST and 6MWT correlated strongly (ρ = 0.82). The frequency of patients with nadir SpO_2 _< 90% was consistent for both tests (κ = 0.82). 1STST was superior to diffusing capacity for carbon monoxide in detecting desaturation during the 6MWT. These findings were similarly stratified according to performance status or dyspnea scale. The 1STST can easily measure exertional desaturation in ILD patients with normal resting blood oxygen levels and is an alternative to the 6MWT.

## Introduction

Interstitial lung diseases (ILDs) are a heterogeneous group of disorders that encompass a large and varied range of conditions^1,2^. In some patients with chronic fibrotic ILDs, a progressive phenotype is comparable to that observed in idiopathic pulmonary fibrosis (IPF), including worsening respiratory symptoms, a decline in lung function, decreased quality of life, and early mortality despite conventional treatment^2,3^. These fibrotic ILDs have recently been termed “progressive fibrosing ILDs” (PF-ILDs)^4^. Predicting the prognosis of patients with ILDs is not only becoming increasingly crucial, but it is also challenging for clinicians.

Although hypoxemia is often absent at rest in patients with ILD, exertional desaturation is more likely seen even in ILD patients with normal oxygen levels at rest^5,6^. For patients with idiopathic interstitial pneumonia (IIP), oxygen desaturation during the 6 min walk test (6MWT) was a strong predictor for mortality^7^. Even in IPF patients with the preserved resting arterial partial pressure of oxygen (PaO2), the presence of exertional desaturation during the 6MWT was a significant prognostic factor for poor survival^8^. The international guidelines recommend that exercise-induced desaturation should be assessed in most patients with ILD as an important prognostic indicator^9^. In Japan, desaturation on 6MWT is included in the disease severity staging system for IIPs^10^. Moreover, the serial 6MWT measurements at certain intervals were proposed as a criterion that may be used in clinical practice to assess disease progression in PF-ILDs^11^.

The 6MWT is a well-established assessment of exercise tolerance and exercise-induced desaturation in various chronic lung diseases^12^. However, the 6MWT is time-consuming and requires a 30 m corridor^12^, which is not common in primary care settings or clinical settings. In fact, even the Swedish IPF Registry, which was implemented in 22 respiratory medicine units across Sweden, reported that only 56% of patients underwent 6MWT^13^. Therefore, a simple exercise tolerance test is needed not only for general practitioners (GPs) but also for pulmonologists. To overcome these spatial and technical limitations, several alternative exercise tests, such as the 1 min sit-to-stand test (1STST), have recently been evaluated^14^. The 1STST requires only a chair and is easily applicable in a small amount of time, making it feasible for use in the primary healthcare setting. In patients with ILD, nadir desaturation during the 1STST was correlated with nadir desaturation during 6MWT^15,16^. However, little is known about 1STST reliability as an alternative tool to the 6MWT in ILD patients with normal resting blood oxygen levels. Therefore, this study aimed to evaluate the usefulness of the 1STST for assessing exertional desaturation in ILD patients with normal resting blood oxygen levels.

## Methods

### Study patients

We retrospectively collected the data of ILD patients with normal resting blood oxygen levels who underwent both 6MWT and 1STST within 1 month at Yamaguchi University Hospital and National Hospital Organization Yamaguchi-Ube Medical Center from October 2020 to May 2021. Normal resting blood oxygen levels were defined as PaO_2_ ≥ 80 Torr, and if PaO2 was not performed, SpO_2_ ≥ 96% was substituted. ILD diagnoses were based on a multidisciplinary discussion. IPF was diagnosed according to the 2018 Clinical Practice Guidelines^17^. IIPs other than IPF were diagnosed based on the 2013 Official Statement of the American Thoracic Society (ATS)/European Respiratory Society (ERS)^1^. CTD-ILD patients fulfilled standard criteria^18–22^. Patients with HP sarcoidosis, and PAP were diagnosed according to the respective criteria^23–25^. The study protocol and its amendments were approved by the ethics committee of the Yamaguchi Medical University (Institutional Review Board number. 2021-057). The requirement for informed consent was waived by the ethics committee because no invasive procedures, interventions, or human samples were used in this retrospective study, and anonymity was secured. This study was compliant with the Japanese Ethical Guidelines for Medical and Health Research Involving Human Subjects^26^, which do not require informed consent from patients enrolled in studies that did not utilize human biological specimens. However, we provided opportunities to the participants to opt out of the study by announcing the study information on the bulletin boards in the hospital and the hospital website.

### Study assessments

Medical records were used to collect data on baseline demographic information. Pulmonary function tests within 3 months of the date of 1STST were also performed. According to the ATS/ERS recommendations, pulmonary function was assessed using the CHESTAC-8800 DN type (Chest Ltd., Tokyo, Japan)^27^. Disease severity was assessed using the sex, age, and physiology (GAP) staging system^28^ and the Japanese Respiratory Society (JRS) severity grading^29^ based on the PaO2 at rest and minimum SpO_2_ during the 6MWT. In addition, Eastern Cooperative Oncology Group (ECOG) performance status (PS) and mMRC dyspnea grades were assessed by the physicians at the time of the 1STST. The ECOG PS is a scale used to assess how a patient’s disease is progressing and how the disease affects the daily living abilities of the patient^30^. It is comprised of five conditions (0 = “normal activity,” 1 = “some symptoms, but no bed rest during daytime,” 2 = “bed rest for less than 50% of daytime,” 3 = “bed rest for more than 50% of daytime,” 4 = “unable to get out of bed”), and good PS was defined as ECOG PS 0 or 1. The mMRC scale comprises five categories that describe the extent of respiratory disability from no disability to almost complete incapacity^31^, and good mMRC was defined as mMRC 0 or 1.

The 6MWT was performed following the international recommendation^12^. In brief, the test was performed on a marked 30 m indoor corridor, and the patients were asked to walk as far as possible within 6 min. The walking distance was recorded at the end of the test. Participants were allowed to take breaks during the test, if necessary. Before and after the test, SpO_2_ and pulse rate were measured using a pulse oximeter. Desaturation was defined as an SpO_2_ < 90%^8^.

The 1STST was performed using a standardized protocol with a standard height chair (46 cm) without armrests positioned against a wall^15,16,32,33^. The test was first demonstrated by the physician and then performed by the patients. The patients were seated upright on a chair with their knees and hips flexed at 90°, feet placed flat on the floor, and their upper limbs folded across the chest without using the hands or arms to assist movement. Patients were asked to perform repetitions of standing upright and then sitting down in the same position at a self-paced motion (safe and comfortable) for as many repetitions as possible in 1 min. Participants were informed of the time when 15 s had remained, but no encouragement was provided during the test. The number of completed repetitions was manually recorded. Measurements of SpO_2_ and pulse rate were performed before and after the 1STST using the same assessment tools as for the 6MWT. Desaturation was defined as SpO_2_ < 90%.

### Statistical analysis

Although no a priori sample size calculation was conducted, a convenience sample of participants was selected based on a previous study^15^. Data are shown as median (interquartile range). Spearman’s rank-order correlation coefficient was used to determine the correlation between the two variables. A Bland-Altman analysis was performed to graphically examine the limits of agreement between the minimum SpO_2_ in the 6MWT and 1STST. Agreement between the ability of the two exercise tests to detect desaturation was assessed using Cohen’s kappa (κ) index. The magnitude of the κ coefficient, which ranges from 0 (without concordance) to 1 (maximum concordance), is usually interpreted as follows: poor (<0.20), weak (0.21–0.40), moderate (0.41–0.60 and), good (0.61–0.80), and very good (0.81–1.00)^34^. Using a receiver operating characteristic (ROC) curve, we determined the cutoff points for identifying the predictive factors for desaturation during the 6MWT. The accuracy of each predictive factor was assessed using the area under the ROC curve (AUC). A *p*-value of less than 0.05 was considered statistically significant. Statistical analyses were performed using JMP Pro ® (version 15.0.0; SAS Institute Inc., Cary, NC, USA).

### Reporting summary

Further information on research design is available in the Nature Research Reporting Summary linked to this article.

## Results

### Patient characteristics

A total of 116 participants were included; 53 had been diagnosed with IPF, 18 with IIPs other than IPF, 38 with collagen tissue disease-associated interstitial lung disease (CTD-ILD), five with hypersensitivity pneumonitis (HP), one with sarcoidosis, and one with autoimmune pulmonary alveolar proteinosis (PAP). The patient characteristics are described in Table 1. The median age of the patients was 72 years. Approximately 90% of the patients had good performance status (PS 0-1), and 70% had good modified Medical Research Council (mMRC 0-1). The median forced vital capacity (FVC) and diffusion lung capacity for carbon monoxide (DLCO) was 86% and 67%, respectively. The median 6MWT distance was 420 m, and the median number of repetitions during the 1STST was 26. No adverse events were observed in the 1STST.

**Table 1:** Clinical characteristics of the study participants.

Variable	Value
Age (years)	72 (64–78)
Sex male	64 (55.2)
Body mass index	23.1 (20.8–24.9)
Smoking status (never/ex/current)	51/61/4 (44.0/52.6/3.4)
Pack years	12.8 (0–36.8)
Underlying disease	
IPF/IIPs other than IPF/CTD-ILD/HP/Sarcoidosis/PAP	53/18/38/5/1/1 (45.7/15.5/32.8/4.3/0.9/0.9)
Comorbidities	
COPD	17 (14.7)
Asthma	13 (11.2)
Heart failure	10 (8.6)
Diabetes mellitus	20 (17.2)
Performance status (0/1/2/3/4)	27/76/13/0/0 (23.3/65.5/11.2/0.0/0.0)
mMRC scale (0/1/2/3/4)	17/64/30/5/0 (14.7/55.2/25.9/4.3/0.0)
Medication	
Use of antifibrotic agents	25 (21.6)
Use of corticosteroid	40 (34.5)
Use of immunosuppressive drugs	23 (19.8)
Use of inhaled corticosteroid	14 (12.1)
Use of bronchodilators	23 (19.8)
PaO2 at rest (Torr)	90.0 (83.8−95.1)
FVC (% predicted)	85.7 (74.5−100.1)
FEV1/FVC ratio (%)	82.1 (77.2–86.3)
DLCO (% predicted)	67.4 (56.7−81.4)
GAP index (points)	3 (2–3)
GAP grade (I/II/III)	94/20/2 (81.0/17.2/1.7)
6MWT	
Distance (m)	420 (353−479)
Baseline SpO_2_ (%)	97 (96−98)
Nadir SpO_2_ (%)	91 (86−94)
Heart rate, pre-test (bpm)	80 (70−87)
Heart rate, post-test (bpm)	113 (99−127)
1STST	
Repetitions (no.)	26 (21−30)
Baseline SpO_2_ (%)	97 (97−98)
Nadir SpO_2_ (%)	93 (88−95)
Heart rate, pre-test (bpm)	80 (70−87)
Heart rate, post-test (bpm)	108 (91−119)

Data are presented as median (interquartile range) or number (%).

*IPF* idiopathic pulmonary fibrosis, *IIPs* idiopathic interstitial pneumonia, *CTD-ILD* collagen tissue disease-associated interstitial lung disease, *HP* hypersensitivity pneumonitis, *PAP* autoimmune pulmonary alveolar proteinosis, *mMRC* modified Medical Research Council, *PaO2* arterial partial pressure of oxygen, *1STST* 1 min sit-to-stand test, *6MWT* 6 min walk test, *bpm* beats per minute, *FVC* forced vital capacity, *DLCO* diffusion lung capacity for carbon monoxide, *SpO*_*2*_ arterial blood hemoglobin saturation.

### Correlation between the SpO_2_ nadir values during the 6MWT and 1STST

**Fig. 1:**
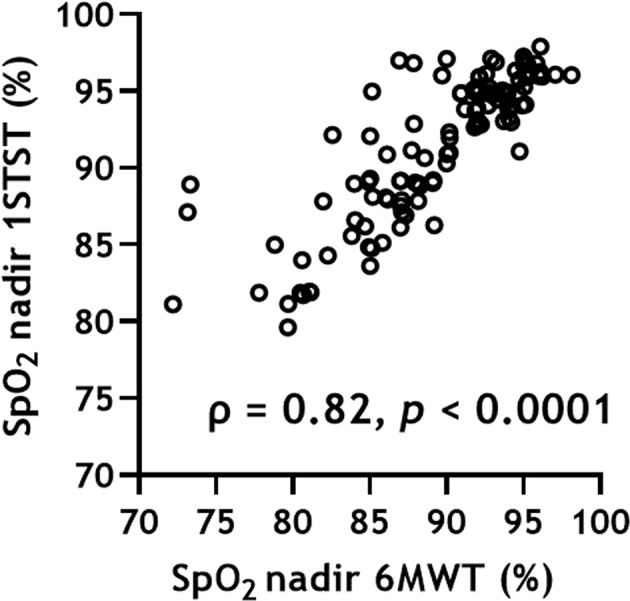
Correlation between the SpO_2_ nadir values during the 6MWT and 1STST. 1STST 1 min sit-to-stand test, 6MWT 6 min walk test, SpO_2_ pulse oxygen saturation.

**Fig. 2:**
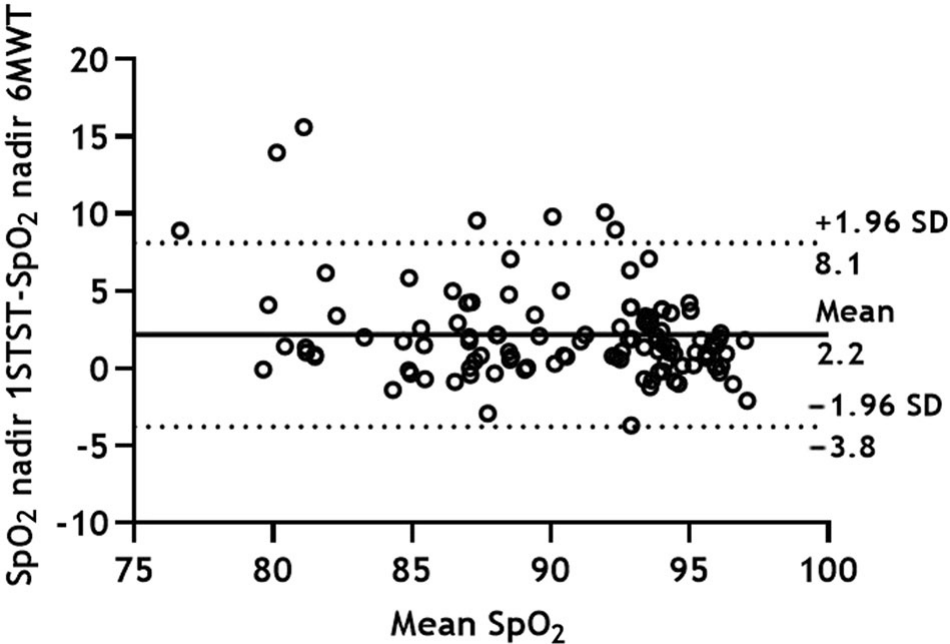
Bland-Altman plot of the difference in SpO_2_ nadir between the 1STST and 6MWT as a function of the mean SpO_2_ nadir in the two tests. The mean difference (solid line) and the limits of the 95% confidence interval (dotted lines) are presented. 1STST 1 min sit-to-stand test, 6MWT 6 min walk test, SpO_2_ pulse oxygen saturation.

We found strong correlations between the pulse oxygen saturation (SpO_2_) nadir values of 6MWT and 1STST (ρ = 0.82, *p* < 0.0001) (Fig. 1). Similar results were obtained when parameters grouped by IPF, PS, and mMRC were analyzed separately. Measurement of agreement between the difference in SpO_2_ nadir between the 1STST and 6MWT resulted in a bias of +2.2 ± 3.0 (95% limit of agreement, −3.8 to 8.1), such as in the Bland-Altman plot (Fig. 2).

### Oxygen desaturation during 1STST and 6MWT

The agreement between oxygen desaturation (nadir SpO_2_ < 90%) during the 1STST and 6MWT is shown in Table 2. The frequency of patients with oxygen desaturation was consistent between the two tests (κ = 0.82; 95% confidence interval, 0.71–0.92). Oxygen desaturation was found in 41 (35%) participants during the 1STST and 51 (43%) participants during the 6MWT. The 1STST induced no oxygen desaturation in 10 (9%) participants, in whom 6MWT did; conversely, the 1STST induced oxygen desaturation in 0 (0%) participants in whom 6MWT did not.

**Table 2:** Oxygen desaturation during 1STST and 6MWT.

	Nadir SpO_2_ < 90% in the 6MWT	
Nadir SpO_2_ < 90% in the 1STST	Yes	No
Yes	41 (35.3)	0 (0.0)
No	10 (8.6)	65 (56.0)

Data are presented as numbers (%).

*1STST* 1 min sit-to-stand test, *6MWT* 6 min walk test, *SpO*_*2*_ pulse oxygen saturation.

### Diagnostic ability to identify desaturation during the 6MWT

**Fig. 3:**
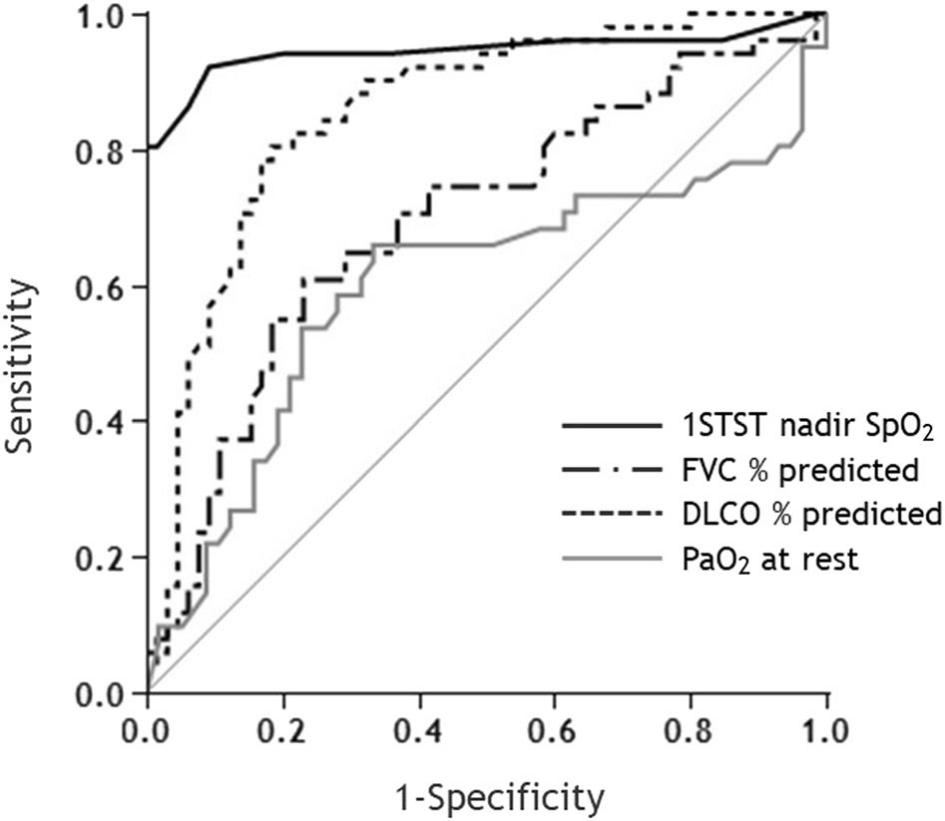
ROC curve to predict the desaturation during the 6MWT. ROC receiver operating characteristic, 1STST 1 min sit-to-stand test, 6MWT 6 min walk test, SpO_2_ pulse oxygen saturation, FVC forced vital capacity, DLCO diffusion lung capacity for carbon monoxide, PaO2 arterial partial pressure of oxygen, AUC area under the curve.

We assessed the diagnostic ability. The study assesses the ability of the 1STST to detect hypoxemia during 6MWT. The study then goes on to compare the diagnostic ability in comparison to other examinations, such as FVC % predicted, DLCO % predicted, and PaO2 at rest (Fig. 3, Table 3). Among these tests, the 1STST had the best ability to detect desaturation during the 6MWT (AUC: 0.94), with a cutoff of 92% and sensitivity and specificity of 92% and 91%, respectively. Moreover, we performed the same analysis in the subgroups of IPF, good PS, and good mMRC. These findings were similar to those observed in the total population (Supplementary Tables 1, 2, 3).

**Table 3:** Diagnostic ability to identify desaturation during 6MWT in all patients.

	AUC (95% CI)	Sensitivity	Specificity	Cutoff value
1STST nadir SpO_2_	0.94 (0.86–0.98)	92%	91%	92%
FVC % predicted	0.69 (0.59–0.78)	61%	77%	80.1
DLCO % predicted	0.86 (0.77–0.91)	80%	82%	66.0
PaO2 at rest	0.60 (0.47–0.71)	66%	67%	89.2 Torr

*SpO*_*2*_ pulse oxygen saturation, *PaO2* arterial partial pressure of oxygen, *1STST* 1 min sit-to-stand test, *6MWT* 6 min walk test, *FVC* forced vital capacity, *DLCO* diffusion lung capacity for carbon monoxide, *AUC* area under the curve, *CI* confidence interval.

## Discussion

In the current study of ILD patients with normal resting blood oxygen levels, we demonstrated that there was a strong correlation between the nadir desaturation during the 1STST with 6MWT and that the 1STST was superior to pulmonary function tests in detecting desaturation during the 6MWT. Moreover, these findings did not differ when patients were stratified by underlying disease, PS, or mMRC scale.

Desaturation on exertion is associated with exercise intolerance, exertional dyspnea, reduced quality of life, and mortality^5,8,35–37^. Since exertional gas exchange abnormalities may appear early in the course of ILD before worsening in DLCO and PaO2, 6MWT is the gold standard for assessing exertional desaturation. For these reasons, in addition to symptoms, pulmonary imaging, and pulmonary physiological indices, such as FVC and DLCO, exertional gas exchange abnormalities have been proposed to assess disease progression in patients with ILDs^9,11^. While the 6MWT is well-validated and reproducible, time-consuming, and the need for a 30 m corridor poses a serious challenge for primary healthcare settings.

The results of this study showed a strong correlation between the nadir desaturation during the 1STST and 6MWT, and the agreement between the two tests on the presence of hypoxemia was extremely high. Moreover, the 1STST SpO_2_ nadir values were ~2% higher than those of the 6MWT. The heart rate post 1STST was also slightly lower than post 6MWT. Although our patient background was older and had a lower BMI (body mass index) and higher diffusion capacity than previously reported, these findings are almost in line with those of previous studies^15,16^. These slight differences in SpO_2_ nadir values and heart rate can be attributed to the fact that the 6MWT may introduce more muscle groups in a systemic exercise and longer exercise duration than the 1STST. In a previous study, the number of patients showing oxygen desaturation during only one of the two tests was not significantly different between the 1STST and 6MWT^15^. Meanwhile, in our results, there was no case of desaturation in 1STST without desaturation in the 6MWT. This discrepancy may be due to the different definitions of desaturation in the previous study and our study. Although desaturation was defined as a fall of 4% or more in the previous study, we defined it as nadir SpO_2_ < 90%, referring to the Japanese classification of disease severity of IIPs (Supplementary Table 4)^8,29^. However, even considering these effects, the results of this study are still very useful.

The 1STST was superior to FVC % predicted, DLCO % predicted, and resting PaO2 in detecting desaturation during the 6MWT. Moreover, these findings did not differ when patients were stratified by an underlying disease, PS, or mMRC scale. There are various tools that predict hypoxemia during the 6MWT in patients with ILD. A recent study demonstrated that resting SpO_2_ ≤ 95% and DLCO ≤ 40% were independently correlated with significant desaturation in 6MWT^38^. This finding is useful in tertiary care respiratory facilities. However, it may be less useful for primary care facilities. To begin with, spirometry is underused in primary care settings, and DLCO is even less underuse. The number of spirometries performed in primary care was surprisingly low. A recent study evaluating referrals from primary and secondary healthcare institutions to tertiary care respiratory specialists revealed that 29% and 4% of the referrals included spirometry and DLCO data, respectively^39^. Patients with mild dyspnea, good general condition, and normal oxygenation are common populations, especially in primary healthcare settings. The finding that 1STST was able to detect hypoxemia during 6MWT with high accuracy in this population reinforces the usefulness of 1STST in primary care facilities.

Since 80% of patients made primary care visits for respiratory complaints 1 year before IPF diagnosis, opportunities exist for the earlier referral on the suspicion of ILDs in primary care^40^. Therefore, GPs play a key role in expediting ILD diagnosis by referring patients to an ILD center or a pulmonologist with expertise in this group of disorders^41^. Both nintedanib and pirfenidone are approved by regulatory agencies worldwide to treat IPF and have received conditional recommendations in the international IPF guidelines^17^. The importance of early initiation of antifibrotic treatment in IPF has received considerable attention in recent years^42–44^. Additionally, based on the results of the INBUILD trial^45^, nintedanib was approved for PF-ILD. The introduction of drugs that effectively slow down disease progression has increased the need for simplified prediction of disease severity and prognosis in patients with ILDs, not only in specialized facilities but also in primary care facilities. Since the presence of exercise-induced hypoxemia is closely related to the disease severity and prognosis, 1STST may be useful for both pulmonologists and GPs in the clinical practice for ILD by providing a simple assessment of exertional hypoxemia.

There are several limitations to this study. First, the sample size was small, and the study design was retrospective. Therefore, the possibility of an unintentional selection bias in patient selection could not be fully excluded. Second, the study patients were characterized by older age and lower BMI. Although the characteristics of patients in the present study are comparable with other IPF studies in Japan^42,43^, these patient characteristics may have influenced the findings, and there could be unmeasured confounders that could impact the results. Third, although all of the patients performed both tests on the same day in the previous reports, 30% of patients performed the 1STST and 6MWT on different days in our study. However, the current findings did not differ between groups where the two tests were conducted on different days and the same day (data not shown). Further prospective studies with larger sample sizes are required to confirm the findings of this study. Furthermore, given the possibility that 1STST may be slightly less load than the 6MWT, the optimal time for the sit-to-stand test needs to be considered in the future.

In conclusion, there was a strong correlation between the nadir desaturation during the 1STST and 6MWT and that the 1STST was superior to pulmonary function tests in detecting hypoxemia during the 6MWT. Our findings highlight the fact that 1STST can measure exertional desaturation in ILD patients with normal resting blood oxygen levels and is a useful alternative tool to the 6MWT.

## Supplementary information

Supplemetary information

REPORTING SUMMARY

## Data Availability

The data generated and/or analyzed during the current study are included in this published article. Additional data are available from the corresponding author upon request.
