# Author Correction: The 1-minute sit-to-stand test to detect desaturation during 6-minute walk test in interstitial lung disease

**DOI:** 10.1038/s41533-022-00274-y

**Published:** 2022-02-25

**Authors:** Keiji Oishi, Kazuto Matsunaga, Maki Asami-Noyama, Tasuku Yamamoto, Yukari Hisamoto, Tetsuya Fujii, Misa Harada, Junki Suizu, Keita Murakawa, Ayumi Chikumoto, Kazuki Matsuda, Haruka Kanesada, Yujiro Kikuchi, Kazuki Hamada, Sho Uehara, Ryo Suetake, Syuichiro Ohata, Yoriyuki Murata, Yoshikazu Yamaji, Kenji Sakamoto, Kosuke Ito, Hisayuki Osoreda, Nobutaka Edakuni, Tomoyuki Kakugawa, Tsunahiko Hirano, Masafumi Yano

**Affiliations:** 1 Department of Medicine and Clinical Science, Graduate School of Medicine, Yamaguchi University, Ube, Japan; 2 Department of Respiratory Medicine and Infectious Disease, Graduate School of Medicine, Yamaguchi University, Ube, Japan; 3 Department of Respiratory Medicine, National Hospital Organization Yamaguchi-Ube Medical Center, Ube, Japan; 4 Department of Pulmonology and Gerontology, Graduate School of Medicine, Yamaguchi University, Ube, Japan

**Keywords:** Respiratory signs and symptoms, Physical examination

Correction to: *npj Primary Care Respiratory Medicine* 10.1038/s41533-022-00268-w, published online 27 January 2022

In Table 2 of this article, the heading for the column labelled “Nadir SpO2 < 90% in the 6MWT” was mistakenly located over the column labelled “Nadir SpO2 < 90% in the 1STST”. The original article has been corrected.

