# Supplementary material for: The 1-minute sit-to-stand test to detect desaturation during 6-minute walk test in interstitial lung disease
Source: NPJ Prim Care Respir Med. 2022 Jan 27;32:5. doi: 10.1038/s41533-022-00268-w (PMC8795411; doi:10.1038/s41533-022-00268-w)
Supplement: Supplementary file 1 — Supplemetary information [file 41533_2022_268_MOESM1_ESM.pdf]

Supplementary Table 1 Diagnostic ability to identify desaturation during the 6MWT in IPF patients

	AUC	Sensitivity	Specificity	Cut-off value
1STST nadir SpO2	0.92 (0.78–0.97)	83%	100%	89%
FVC % predicted	0.76 (0.61–0.87)	63%	88%	82.7
DLCO % predicted	0.91 (0.80–0.96)	83%	92%	65.6
PaO2 at rest	0.64 (0.46–0.78)	56%	82%	86.5 Torr

PaO2, arterial partial pressure of oxygen; 1STST, 1-min sit-to-stand test; 6MWT, 6-min walk test; FVC, forced vital capacity; DLCO, diffusion lung capacity for carbon monoxide; AUC, area under the curve; CI, confidence interval.

Supplementary Table 2 Diagnostic ability to identify desaturation during the 6MWT in good PS

	AUC (95% CI)	Sensitivity	Specificity	Cut-off value
1STST nadir SpO2	0.93 (0.83–0.97)	90%	90%	92%
FVC % predicted	0.70 (0.58–0.79)	62%	79%	80.1
DLCO % predicted	0.84 (0.74–0.90)	76%	82%	66.0
PaO2 at rest	0.57 (0.43–0.70)	64%	66%	89.0 Torr

PaO2, arterial partial pressure of oxygen; 1STST, 1-min sit-to-stand test; 6MWT, 6-min walk test; FVC, forced vital capacity; DLCO, diffusion lung capacity for carbon monoxide; AUC, area under the curve; CI, confidence interval.

Supplementary Table 3 Diagnostic ability to identify desaturation during the 6MWT in good mMRC

	AUC (95% CI)	Sensitivity	Specificity	Cut-off value
1STST nadir SpO2	0.90 (0.75–0.97)	85%	91%	92%
FVC % predicted	0.68 (0.54–0.80)	58%	82%	80.1
DLCO % predicted	0.86 (0.74–0.92)	69%	89%	61.3
PaO2 at rest	0.62 (0.44–0.78)	60%	83%	86.5 Torr

PaO2, arterial partial pressure of oxygen; 1STST, 1-min sit-to-stand test; 6MWT, 6-min walk test; FVC, forced vital capacity; DLCO, diffusion lung capacity for carbon monoxide; AUC, area under the curve; CI, confidence interval.

Supplementary Table 4 Definition of the Japanese severity staging systems for Idiopathic interstitial pneumonias

PaO2	SpO2 < 90% at the end of 6MWT	Stage
≥80 Torr	No	I
	Yes	I
70-79 Torr	No	II
	Yes	III
60-69 Torr	No	III
	Yes	IV
<60 Torr	No	IV
	Yes	IV

PaO2, arterial partial pressure of oxygen; SpO2, pulse oxygen saturation; 6MWT, 6-min walk test.
