# Supplementary material for: The 1-minute sit-to-stand test to detect desaturation during 6-minute walk test in interstitial lung disease
Source: NPJ Prim Care Respir Med. 2022 Jan 27;32:5. doi: 10.1038/s41533-022-00268-w (PMC8795411; doi:10.1038/s41533-022-00268-w)
Supplement: Supplementary file 2 — REPORTING SUMMARY [file 41533_2022_268_MOESM2_ESM.pdf]

nature portfolio

Corresponding author(s): Keiji Oishi

Last updated by author(s): 2021-Aug-15

Reporting Summary

Nature Portfolio wishes to improve the reproducibility of the work that we publish. This form provides structure for consistency and transparency in reporting. For further information on Nature Portfolio policies, see our Editorial Policies and the Editorial Policy Checklist.

Statistics

For all statistical analyses, confirm that the following items are present in the figure legend, table legend, main text, or Methods section.

n/a	Confirmed	
☐	☒	The exact sample size (n) for each experimental group/condition, given as a discrete number and unit of measurement
☐	☒	A statement on whether measurements were taken from distinct samples or whether the same sample was measured repeatedly
☐	☒	The statistical test(s) used AND whether they are one- or two-sided<br>*Only common tests should be described solely by name; describe more complex techniques in the Methods section.*
☐	☒	A description of all covariates tested
☐	☒	A description of any assumptions or corrections, such as tests of normality and adjustment for multiple comparisons
☐	☒	A full description of the statistical parameters including central tendency (e.g. means) or other basic estimates (e.g. regression coefficient) AND variation (e.g. standard deviation) or associated estimates of uncertainty (e.g. confidence intervals)
☐	☒	For null hypothesis testing, the test statistic (e.g. F, t, r) with confidence intervals, effect sizes, degrees of freedom and P value noted<br>*Give P values as exact values whenever suitable.*
☒	☐	For Bayesian analysis, information on the choice of priors and Markov chain Monte Carlo settings
☒	☐	For hierarchical and complex designs, identification of the appropriate level for tests and full reporting of outcomes
☐	☒	Estimates of effect sizes (e.g. Cohen's d, Pearson's r), indicating how they were calculated

Our web collection on statistics for biologists contains articles on many of the points above.

Software and code

Policy information about availability of computer code

Data collection	Data collection were performed using JMP Pro ® (version 15.0.0; SAS Institute Inc., Cary, NC, USA).
Data analysis	Data analyses were performed using JMP Pro ® (version 15.0.0; SAS Institute Inc., Cary, NC, USA).

For manuscripts utilizing custom algorithms or software that are central to the research but not yet described in published literature, software must be made available to editors and reviewers. We strongly encourage code deposition in a community repository (e.g. GitHub). See the Nature Portfolio guidelines for submitting code & software for further information.

Data

Policy information about availability of data

All manuscripts must include a data availability statement. This statement should provide the following information, where applicable:

- Accession codes, unique identifiers, or web links for publicly available datasets
- A description of any restrictions on data availability
- For clinical datasets or third party data, please ensure that the statement adheres to our policy

The data generated and/or analyzed during the current study are included in this published article. Additional data are available from the corresponding author upon request.

Field-specific reporting

Please select the one below that is the best fit for your research. If you are not sure, read the appropriate sections before making your selection.

☒ Life sciences ☐ Behavioural & social sciences ☐ Ecological, evolutionary & environmental sciences

For a reference copy of the document with all sections, see nature.com/documents/nr-reporting-summary-flat.pdf

Life sciences study design

All studies must disclose on these points even when the disclosure is negative.

Sample size	Although no a priori sample size calculation was conducted, a convenience sample of participants was selected based on a previous study.
Data exclusions	No data ware excluded.
Replication	n/a
Randomization	n/a
Blinding	n/a

Reporting for specific materials, systems and methods

We require information from authors about some types of materials, experimental systems and methods used in many studies. Here, indicate whether each material, system or method listed is relevant to your study. If you are not sure if a list item applies to your research, read the appropriate section before selecting a response.

Materials & experimental systems

n/a	Involved in the study	
☒	☐	Antibodies
☒	☐	Eukaryotic cell lines
☒	☐	Palaeontology and archaeology
☒	☐	Animals and other organisms
☐	☒	Human research participants
☒	☐	Clinical data
☒	☐	Dual use research of concern

Methods

n/a	Involved in the study	
☒	☐	ChIP-seq
☒	☐	Flow cytometry
☒	☐	MRI-based neuroimaging

Human research participants

Policy information about studies involving human research participants

Population characteristics	We retrospectively collected the data of ILD patients with normal resting blood oxygen levels who underwent both 6MWT and 1STST within 1 month at Yamaguchi University Hospital and National Hospital Organization Yamaguchi-Ube Medical Center from October 2020 to May 2021. Normal resting blood oxygen levels were defined as PaO2 ≥80 Torr, and if PaO2 was not performed, SpO2 ≥96% was substituted.
Recruitment	The requirement for informed consent was waived by the ethics committee because no invasive procedures, interventions, or human samples were used in this retrospective study, and anonymity was secured. This study was compliant with the Japanese Ethical Guidelines for Medical and Health Research Involving Human Subjects, which do not require informed consent from patients enrolled in studies that did not utilize human biological specimens. However, we provided opportunities to the participants to opt out of the study by announcing the study information on the bulletin boards in the hospital and the hospital website.
Ethics oversight	The study protocol and its amendments were approved by the ethics committee of the Yamaguchi Medical University (Institutional Review Board number. 2021-057).

Note that full information on the approval of the study protocol must also be provided in the manuscript.
